# Phase I study of an active immunotherapy for asymptomatic phase Lymphoplasmacytic lymphoma with DNA vaccines encoding antigen-chemokine fusion: study protocol

**DOI:** 10.1186/s12885-018-4094-2

**Published:** 2018-02-13

**Authors:** Sheeba K. Thomas, Soung-chul Cha, D. Lynne Smith, Kun Hwa Kim, Sapna R. Parshottam, Sheetal Rao, Michael Popescu, Vincent Y. Lee, Sattva S. Neelapu, Larry W. Kwak

**Affiliations:** 1 Department of Lymphoma/Myeloma, Division of Cancer Medicine, The University of Texas MD Anderson Cancer Center, Houston, TX 77030 USA; 2 Department of Stem Cell Transplantation and Cellular Therapy, The University of Texas MD Anderson Cancer Center, Houston, TX 77030 USA; 3 Toni Stephenson Lymphoma Center, Department of Hematology and Hematopoietic Stem Cell Transplantation, Beckman Research Institute of City of Hope, Duarte, CA 91010 USA

**Keywords:** DNA vaccine, Personalized medicine, Lymphoma, Phase I, Idiotype, Immune response

## Abstract

**Background:**

There is now a renewed interest in cancer vaccines. Patients responding to immune checkpoint blockade usually bear tumors that are heavily infiltrated by T cells and express a high load of neoantigens, indicating that the immune system is involved in the therapeutic effect of these agents; this finding strongly supports the use of cancer vaccine strategies. Lymphoplasmacytic lymphoma (LPL) is a low grade, incurable disease featuring an abnormal proliferation of Immunoglobulin (Ig)-producing malignant cells. Asymptomatic patients are currently managed by a “watchful waiting” approach, as available therapies provide no survival advantage if started before symptoms develop. Idiotypic determinants of a lymphoma surface Ig, formed by the interaction of the variable regions of heavy and light chains, can be used as a tumor-specific marker and effective vaccination using idiotypes was demonstrated in a positive controlled phase III trial.

**Methods:**

These variable region genes can be cloned and used as a DNA vaccine, a delivery system holding tremendous potential for streamlining vaccine production. To increase vaccination potency, we are targeting antigen-presenting cells (APCs) by fusing the antigen with a sequence encoding a chemokine (MIP-3α), which binds an endocytic surface receptor on APCs. Asymptomatic phase LPL is an excellent model to test our vaccine since patients have not received chemotherapeutics that interfere with innate immune function and have low tumor burden. We are evaluating the safety of this next-generation DNA vaccine in a first-in-human clinical trial currently enrolling asymptomatic LPL patients. To elucidate the mode of action of this vaccine, we will assess its ability to generate tumor-specific immune responses and examine changes in the immune profile of both the peripheral blood and bone marrow.

**Discussion:**

This vaccine could shift the current paradigm of clinical management for patients with asymptomatic LPL and inform development of other personalized approaches.

**Trial registration:**

ClinicalTrials.gov identifier NCT01209871; registered on September 24, 2010.

## Background

Development of a vaccine against human malignancies has been frustrated by the difficulty of identifying tumor-specific antigens which would distinguish tumor cells from normal cells and which could be used to induce the host’s immune system to reject cells bearing that antigen. The problem of tumor-specific antigen identification is solved in B-cell malignancies, which are clonal proliferations of cells expressing a single cell surface immunoglobulin (Ig) molecule with unique highly specific, heavy and light chain variable regions. These recurring sequences form unique antigen-recognition sites, and contain determinants that can themselves be recognized as antigens, or idiotypes, which then serve as tumor-specific marker for the malignant clone.

Animal and human studies have demonstrated the utility of the Ig idiotype as a tumor-specific antigen [1–3]. Active immunization against idiotypic determinants on malignant B cells has resulted in idiotype tumor resistance in a number of syngeneic models [4–13]. These results provided the rationale for testing autologous tumor-derived idiotypic surface Ig (Id) as a therapeutic “vaccine” against human B-cell lymphoma [14].

The clinical efficacy of therapeutic Id vaccination in follicular lymphoma (FL) has been tested in a randomized double-blind placebo controlled multicenter Phase III trial initiated by the National Cancer Institute, and subsequently sponsored by Biovest International Inc. Patients with previously untreated advanced stage FL were treated with PACE chemotherapy regimen until clinical remission. Patients achieving complete remission were randomized at a ratio of 2:1 to receive Id-KLH plus GM-CSF or KLH plus GM-CSF. The primary endpoint for this trial was disease free survival (DFS) [15]. After a median follow-up of 56.6 months (range 12.6 - 89.3 months), median time to relapse after randomization for the Id-KLH/GM-CSF arm was 44.2 months, versus 30.6 months for the control arm, suggesting benefit for this vaccine (*p*-value = 0.045; HR = 1.6) [16].

Manufacturing patient-specific idiotype protein for the Phase III trial was expensive and required 3-6 months for each patient. In contrast, DNA vaccines are simple and easy to produce. In vivo expression of foreign genes encoding the tumor antigen by DNA vaccination requires only that the gene is cloned into an expression cassette under eukaryotic or viral regulatory element control; the cassette is then either injected in solution intramuscularly or intradermally or delivered into the epidermis by particle mediated bombardment of DNA-coated gold particles (gene gun). Current antibody engineering makes it possible to readily identify and clone Ig variable genes, including specific B-cell malignancy V-genes [17, 18], and to combine these into a single chain Fv (scFv) format which is a single polypeptide of VH and VL connected in frame by a 15 amino acid linker.

The scFv required an adjuvant to render it immunogenic in mouse studies [19–21]. Therefore, we fused the scFv to proinflammatory chemokines. Chemokines are key effector molecules regulating the selective trafficking of professional antigen presenting cells (APC), including dendritic cells (DC), through peripheral tissues to reach lymph nodes [22, 23]. We have shown that mice immunized by bombardment of gold particles coated with plasmids encoding either of two chemokines – interferon inducible protein 10 (IP-10), or monocyte chemotactic protein 3 (MCP3) – fused with scFv, but not scFv alone, mounted protective antitumor immunity against a large tumor challenge (20 times the minimum lethal dose). Moreover, the DNA fusions induced effector CD4+ and CD8+ T cells, which were required for protection. Finally, the level of protection was greater than or equal to that of the prototype Id-KLH protein in both tumor models [24]. We further demonstrated that intact secretion leader sequences and the chemokine receptor binding site of MCP3 were required for this activity [25]. Taken together, this strongly suggested that antitumor immunity was triggered by the targeting of APC for chemokine receptor-mediated uptake of antigen, rather than recruitment of APC by the chemokine.

In this study, we aim to translate the knowledge gained in FL into a useful treatment for patients with lymphoplasmacytic lymphoma (LPL). Our hypothesis is that anti-tumor immunity can be triggered by targeting APC in vivo with a chemokine-tumor antigen fusion protein. In this clinical trial, we intend to use recombinant plasmid DNA encoding a fusion protein consisting of autologous lymphoma scFv and the human CCL20 (macrophage inflammatory protein-3 alpha - MIP-3α) chemokine. The MIP-3α receptor, CCR6, is preferentially expressed on CD1a + Langerhans cells, CD34-derived immature dendritic cells, and B cells [26]. Following intradermal injection of the recombinant plasmid, the secreted fusion protein should be efficiently bound and internalized through CCR6 on the Langerhans cells and immature dendritic cells. The targeted delivery of this fusion protein to these professional antigen-presenting cells, and subsequent processing and presentation, can break the tolerance to generate an immune response against the idiotype. The activated idiotype-specific immunity would then serve as the main force to eradicate the antigen-expressing B-cell lymphoma cells.

LPL is a low grade B-cell lymphoproliferative disorder characterized by bone marrow infiltration with lymphoplasmacytic cells, together with a monoclonal gammopathy, as defined by the Revised European-American Lymphoma and WHO classification systems [27]. Current recommendations are that patients with asymptomatic LPL should be observed, as they may have a lengthy indolent course not requiring therapy [28, 29]. Treatment for systemic disease elicits high overall response rates. However, complete responses are infrequent, eventual relapse from disease is inevitable, and LPL remains incurable. Studies performed in patients with FL targeted those patients with minimal residual disease following induction chemotherapy for symptomatic disease. However, the nucleoside analogs and/or alkylating agent based therapy often used in patients with symptomatic LPL affects the function of their T cells. Since proper T cell function is indispensable to inducing a response to vaccine therapy, this study will instead enroll patients in the asymptomatic phase of LPL, who should have intact T cell function.

We expect to find that higher immunosuppressive cell counts in pre-vaccination samples are associated with lower quality and magnitude of the tumor/peptide-specific T cell responses. We also expect that effector T-cell responses are likely to be higher in patients receiving the higher dose of the vaccine compared with the lower dose. Further, we hypothesize that post-vaccination we will observe increased CD8^+^ (and CD4^+^) to FoxP3^+^ ratios and decreases in PD-1 and CTLA-4 expression by T cells. Finally, post-vaccination we expect to see an increased number and density of effector T cells interacting with LPL tumor cells. These results could inform the future development of new molecular biomarkers.

While the statistical power of the immunogenicity analysis will be limited by the small sample size; preliminary biologic activity readouts are likely to be hypothesis-generating. The immunogenicity data may also provide guidance on alternative vaccine formulations using other adjuvants or combinations with immune checkpoint inhibitors in future clinical trials, particularly if decreases in immune regulatory molecule expression are observed post-vaccination, or if robust effector immune responses are observed, but with only modest clinical responses.

### Objectives

The primary objectives are to i) evaluate the safety of using a novel lymphoma DNA vaccine encoding MIP3α-fused lymphoma idiotype in single chain format, and ii) to determine the maximum tolerated dose (MTD) of the vaccine in subjects with LPL. A secondary objective is to assess the ability of the vaccine to generate tumor-specific cellular and humoral immune responses.

## Methods

### Study design

This study is a prospective clinical investigation designed to generate safety data. It is an open-label, phase I trial designed to determine the safety and tolerability of intradermal administration of patient-specific scFv-CCL20 DNA vaccine in patients with asymptomatic LPL. Patients will receive a series of 3 vaccinations at 4-week intervals (weeks 0, 4 and 8). The 2 pre-defined dose levels – Cohort 1 (500 μg DNA vaccine) and Cohort 2 (2500 μg DNA vaccine) – were chosen based on the pre-clinical experience [24, 25].

All patients will either undergo bone marrow aspiration of 10 ml or lymph node harvest prior to starting treatment to obtain cells to prepare their vaccine. Once the vaccine for a given patient is available, he or she will receive 3 doses of DNA vaccine encoding autologous lymphoma scFv- human CCL20 (MIP-3α) fusion protein.

Enrollment to Cohorts 1 and 2 will follow a standard 3 + 3 statistical design, and will thus require a maximum of 12 patients, and shall be staggered to ensure no patient is administered the vaccine until any previously exposed patient has completed a minimum of 48 h of safety follow-up after receiving the first DNA vaccine administration. Prior to advancing dose levels, a cohort summary will be completed and submitted to the IND office medical monitor for review and approval to proceed to the next cohort.

If patients have progressive disease that requires chemotherapy or radiotherapy treatment before or while receiving vaccination, vaccination will either not begin or will be stopped, and the patient will be taken off study. Patients will be vaccinated intradermally and the injection sites will be rotated between the thighs. Patients will be observed in CTRC for 2 h after vaccine administration; vital signs will be taken every 15 min during the first hour and every 30 min during the second hour. The use of non-steroidal anti-inflammatory drugs (NSAIDs) and/or steroids should be avoided during the vaccination. Should NSAIDs or steroids be required for unrelated medical conditions for a course exceeding 2 weeks, the patient will be taken off the study. Patients will receive a list of common aspirin containing products to avoid at the time of study entry. Any local skin reactions will be carefully noted and scored for erythema, induration, pain and disruption of the barrier surface.

Toxicities will be graded according to the NCI Common Toxicity Criteria v4.0. No further vaccinations will be given to patients who develop grade 3 or 4 hypersensitivity reactions or grade 3 injection site reactions. No dose modification will be made for grade 3 fever. For grade 4 fever, subsequent vaccinations will be administered at 50% of the original dose level. For all other grade 3 or 4 toxicity reactions, no further vaccinations will be given if in the opinion of the investigator the toxicity is related to the vaccine administration.

### Patients

Adult (age ≥ 18 years) patients with asymptomatic phase, previously untreated LPL with surface IgG, IgA or IgM phenotype tissue diagnosis and a monoclonal heavy and light chain as determined by flow cytometry who are able to provide informed consent will be eligible for this study [30]. All primary diagnostic lymph node and/or bone marrow biopsies will be reviewed at the University of Texas M.D. Anderson Cancer Center (UTMDACC). Patients must provide a lymph node sample of at least 1.5 cm in the long axis, or a bone marrow aspiration sample providing at least 5 million CD20 and/or CD38+ cells (approximately 10 ml). Patients mush also have a good performance status (ECOG 0 or 1), and be able to attend clinic for adequate follow-up for the period that the protocol requires. In addition patients must have adequate kidney and liver function, defined by serum creatinine ≤1.5 mg/dl and a creatinine clearance ≥ 30 ml/min, and total bilirubin ≤1.5 mg/dl unless believed secondary to Gilbert’s disease, and AST/ALT ≤2 x upper limit of normal.

Female subjects must be either post-menopausal or surgically sterilized or willing to use an acceptable method of birth control (i.e., a hormonal contraceptive, intra-uterine device, diaphragm with spermicide, condom with spermicide, or abstinence) for the duration of the study and for 30 days after the last vaccination has been administered. Male subjects should agree to use an acceptable method for contraception for the duration of the study.

Patients will be excluded from the study if they have HIV, Hepatitis B and/or Hepatitis C infection; a previous history of malignancy within the last 5 years except curatively treated squamous or basal cell carcinoma of the skin or curatively treated carcinoma in-situ of other organs; have any medical or psychiatric condition that in the opinion of the principal investigator would compromise the patient’s ability to tolerate this treatment; have New York Heart Association Class 3 or 4 disease; have a history of autoimmune diseases except for Hashimoto’s thyroiditis; or have a positive ANA and/or anti-dsDNA antibodies. Additionally, pregnant or lactating females are excluded.

All patients will be registered in the Clinical Oncology Research System (CORe). All patients who have asymptomatic LPL will be seen by members of the UTMDACC Department of Lymphoma and Myeloma, and tracked by the research nurse/study coordinator in charge of this study. Participation on this protocol will be discussed with patients deemed eligible. Formal screening for eligibility will occur if informed consent is received. The informed consent document is included in the Supplementary Data. For eligible patients not enrolled on the study, a Health Insurance Portability and Accountability Act (HIPAA) compliant pre-enrollment and enrollment log will be maintained in CORe to track reason(s) for lack of enrollment. The patient’s entry date on protocol will be the day the patient is registered in CORe. The treatment start date will be the day the first dose of vaccine is administered to the patient.

Patients will be removed from protocol for any of the following reasons: adverse event(s) occur that in the judgment of the investigator, may cause severe or permanent harm or which rule out continuation of study drug; the patient declines further therapy, experiences progression of LPL that requires initiation of systemic therapy to control symptoms, or has an inadequate number of biopsy cells (< 1.0 × 10^9^) for vaccine manufacture or there is an unforeseen vaccine manufacturing failure. Additionally a patient will be removed from protocol if it is deemed in their best interest, in which case the Principal Investigator should be notified, and the reasons for withdrawal should be noted in the flow sheet.

### Study drug

The scFv-CCL20 plasmid DNA vaccine is prepared from the VH and VL lymphoma immunoglobulin variable regions of each patient’s tumor cells in three cloning steps [31]. First, the individual VH and VL are cloned by RT-PCR using consensus primers. The mature V region sequences are then cloned in-frame with a short linker to yield a single chain antibody gene (scFv). Lastly, the scFv gene sequence is cloned in-frame to the 3′ end of the human CCL20 (MIP-3α) gene via a spacer sequence. At each stage the DNA sequences are verified. The plasmid DNA is then amplified in *E coli,* and subsequently purified from *E coli* according to Good Manufacturing Practices (GMP) standards and tested for sterility and endotoxin contamination prior to its use in any patient. The entire vaccine preparation procedure takes 12 months and is conducted at FUJIFILM Diosynth Biotechnologies U.S.A., Inc., GMP facility.

The drug is formulated on a 0.5 ml basis with patient-specific scFv-CCL20 plasmid DNA vaccine. Each patient-specific formulation will have 500 μg or 2500 μg of the plasmid DNA depending on the cohort assignment of the patient. Each vial of patient-specific vaccine will be labeled with the following information: scFv-CCL20 DNA vaccine, patient last name and first initial, patient-specific lot, final volume, storage conditions, and fill date. The protocol number, drug strength, expiration date, and the statement, “Caution: New drug – Limited by Federal law to investigational use,” will also appear on the vaccine vial label.

Prior to administration the vaccine will be stored at − 20 °C. Immediately prior to dosing, the contents of the vial will be thawed to room temperature. After gentle agitation, the vial contents should be drawn up using a filling adaptor attached to a syringe for the Tropis™ intradermal needle free injection device. After the contents of the vial have been drawn up, the syringe assembly will be inserted into the needle free injector. The vial adaptor will then be removed, and the vaccine will be administered by needle-free injection device for intradermal injection in the thighs. The syringe should be refrigerated at 2 °C to 8 °C and the vaccine will be administered in a total volume of 0.5 mL. Due to the 0.1 ml volume limit of the Tropis™ intradermal needle free injection device, the total volume of 0.5 ml will be administered in 5 injections.

The vaccine is expected to result in minimal toxicities. The most likely anticipated reactions include local erythema and induration at the injection sites and transient flu-like symptoms. No known oncogenic or immunomodulatory sequences are detected in the plasmid.

### Study procedures

Table 1 summarizes the schedule of study procedures. Research eligibility evaluations should be performed within 1 year prior to the start of therapy. For correlative studies 10 ml peripheral blood for serum and 60 ml for PBMC isolation should also occur during this 12 months. For patients who have measurable serum monoclonal protein, a portion of this serum sample will be used to isolate idiotype M protein for immunologic assays. Importantly, in the year prior to starting therapy, all patients must undergo aspiration of 10 ml of bone marrow tissue for routine morphological classification, and immunophenotypic characterization. A second 15 ml bone marrow aspirate sample will be collected from the contralateral side. This bone marrow aspiration may be repeated once to yield sufficient tumor cells to clone the lymphoma Ig VH and VL chains for vaccine manufacture, and to perform immune monitoring studies. Once plasmid constructs have been developed, the constructs will be shipped to the Good Manufacturing Practice Laboratory of FUJIFILM Diosynth Biotechnologies U.S.A., Inc., for clinical grade manufacturing of each patient-specific vaccine.

**Table 1:** Study calendar

Data Collection Instrument	Screening Eligibility	Baseline	Vaccine 1	Vaccine 2	Vaccine 3	Post Vaccine Follow-up 1	Post Vaccine Follow-up 2	Blood Work 1	Post Vaccine Follow-up 3	Post Vaccine Follow-up 4	Blood Work 2	Post Vaccine Follow-up 5	Post Vaccine Follow-up 6	Blood Work 3	Post Vaccine Follow-up 7	Blood Work 4	End of Study/Progression
Study ID & ICF Information	X																
Demographics	X																
Screening Eligibility	X																
H&P, Vitals, ECOG	X	X	X	X	X	X	X	X	X	X	X	X	X	X	X	X	X
Physical Exam	X	X	X	X	X	X	X	X	X	X	X	X	X	X	X	X	X
Concomitant Medications	X																
Chemistries	X	X	X	X	X	X	X		X	X		X	X		X		X
Hematology, PT/PTT	X	X	X	X	X	X	X		X	X		X	X		X		X
Lymphoma Labs Serum	X	X	X	X	X	X	X		X	X		X	X		X		X
Lymphoma Labs Urine	X	X	X	X	X	X	X		X	X		X	X		X		X
ANA ds DNA	X	X	X	X	X	X	X		X	X		X	X		X		X
Infectious Disease Panel	X																
EKG/Imaging	X	X															
Bone Marrow Biopsy	X																
Vaccine Therapy			X	X	X												
Disease Assessment				X	X	X	X		X	X		X	X		X		X
Correlative Lab Draw	X		X	X	X	X		X			X			X		X	X
Off Study Evaluation			X	X	X	X	X	X	X	X	X	X	X	X	X	X	X
Long term follow-up																	X
AE/SAE CTCAE v4		X	X	X	X	X	X	X	X	X	X	X	X	X	X	X	X

Lymph Node Harvest/Biopsy – in patients whose LPL presents primarily as lymphadenopathy, and who lack significant bone marrow involvement (< 10%), a safely accessible lymph node, measuring at least 1.5 cm in the long axis, will be harvested in lieu of the bone marrow aspirate and collected in sterile saline. One third of the specimen will be used for morphological classification and immunophenotypic characterization. Two-thirds of the specimen will be used to provide starting material for manufacture of the vaccine, and sent to the UTMDACC Lymphoma/Myeloma Core laboratory and assigned a unique accession number. Should the first lymph node sample not provide sufficient tumor cells for the vaccine production and immunologic assays, a second excisional lymph node biopsy may be performed to reach the needed yield.

If pathologic lymphadenopathy or hepatosplenomegaly were noted on CT scan imaging during the Research Eligibility Evaluation (REE) OR there is clinical concern for development of enlarged lymph nodes/organomegaly compared with scans performed for the REE, the following restaging imaging will also be performed within 1 week of receiving the 1st dose of vaccine to serve as the baseline for subsequent comparison; CT scans of Neck, Chest, Abdomen and Pelvis. Unless clinically indicated, this imaging will only be obtained if it has been ≥6 weeks since scans were performed for the REE.

On study evaluations are indicated in Table 1, and should occur within 48 h prior to each vaccination. Toxicities documented on the day of the first vaccination (prior to receipt of the vaccine) will serve as the baseline assessment. For immunological studies 10 ml peripheral blood for serum and 30 ml for peripheral blood mononuclear cells (PBMC) isolation will be collected within 2 days prior to day 1 of each vaccination. There is a +/− 5 business day window for all the following events: post-vaccine therapy evaluations at 4 weeks after the last vaccination and every 2 months thereafter for a year; re-staging CT scans of the neck, chest, abdomen and pelvis at 4 weeks after the final vaccination and every 6 months thereafter for a year; collection of 10 ml peripheral blood for serum for storage and 60 ml for PBMC isolation at 4 weeks after the last vaccination and every 4 months for 1 year thereafter for immunological studies.

#### Specimen processing

Blood and tissue specimens collected in the course of this research project may be banked and used in the future to investigate new scientific questions related to this study and for basic studies of lymphoma biology in vitro. However, this research may only be done if the risks of the new questions were covered in the consent document. If new risks are associated with the research (e.g., analysis of germ line genetic mutations) the principal investigator must amend the protocol and obtain informed consent from all research subjects.

All peripheral blood samples will be sent promptly to the Lymphoma/Myeloma Core laboratory, UTMDACC. Red top tubes will be spun down and serum divided into 1 ml aliquots and frozen. PBMCs will be isolated prior to freezing by Ficoll-Hypaque centrifugation using standard protocols.

#### Assay for serum tumor-specific antibody

Serum tumor-specific antibody will be assayed by flow cytometry. Serial dilutions of pre-immune and hyperimmune serum samples from each patient will be mixed with autologous tumor cells (or idiotype monoclonal protein when available) or isotype-matched irrelevant tumor cells (or monoclonal protein of irrelevant specificity) from a different patient. Bound anti-tumor antibody is detected with FITC labeled goat anti-human light-chain antibodies directed against the light chain not present in the immunoglobulin idiotype (Caltag Laboratories, South San Francisco). A response is considered positive when a four-fold rise in the bound antibody titer is observed compared to the pre-vaccine serum and the isotype matched irrelevant tumor used as specificity controls.

#### Assay of precursor frequency of tumor-specific cytotoxic T-lymphocytes

The precursor frequency of tumor-specific cytotoxic T-lymphocytes will be determined by modified interferon gamma (IFNγ) ELISPOT assay as described previously [32]. A three-fold rise in the frequency of tumor-reactive T cell precursors in the post-vaccine PBMC sample compared to the pre-vaccine sample will be considered positive, with a minimum precursor frequency of 1 in 80,000 cells if the precursor frequency in the pre-vaccine sample is zero [33].

#### Assay for anti-MIP-3α antibodies

Anti-MIP-3α antibodies will be assessed by direct enzyme-linked immunosorbent assay (ELISA). Pre-immune and hyperimmune serum samples from each patient will be diluted over wells of a microtiter plate that are coated with human MIP-3α. Bound antibody is detected with horseradish peroxidase-goat antihuman IgG (Caltag Laboratories, South San Francisco). A four-fold rise in the bound antibody titer in the post-vaccine serum vs pre-vaccine serum and an irrelevant human chemokine used as specificity control constitutes a positive response.

Disease response and relapse/progression will be assessed according to the criteria in the Update on Recommendations for Assessing Response from the Third International Workshop on Waldenstrom’s Macroglobulinemia [34].

### Statistical plan

DLT is defined as follows: ≥ grade 2 allergic reaction, ≥ grade 2 autoimmune reaction and any grade 3 or 4 toxicity except for fever, grade 4 fever which subsequently requires 50% dose reduction. Applying the 3 + 3 design [35], the first cohort of 3 patients will be treated at dose level 1 and evaluated for DLT at the end of the first cycle (4 weeks). Following the standard 3 + 3 design, the MTD is defined as the highest dose level in which 6 patients have been treated with less than 2 instances of DLT [35]. Given 2 predefined dose levels, it is anticipated that up to 12 eligible patients are required for this dose-finding phase I trial.

If greater than 1 out of 3 patients or 1 out of 6 patients experience DLT at the lower dose level, this dose will be considered too toxic and the protocol will be stopped.

Toxicity type and severity will be summarized by frequency tables.

The secondary endpoint of immune response will estimated using the intent-to-treat population. Immune response will be defined as at least a three-fold rise in the frequency of tumor-reactive precursor T-cells in the 12 weeks post-vaccine PBMC sample compared with the pre-vaccine sample, or a minimum precursor frequency of 1 in 80,000 cells post vaccination if the precursor frequency in the pre-vaccine sample is zero.

### Safety and adverse events

All patients must have signed an Informed Consent and a completed on-study confirmation of eligibility form before entering on the study. Complete records will be maintained on REDCap (Research Electronic Data Capture) and Clinical Oncology Research System (CORe). REDCap is an electronic data capture tool hosted at UTMDACC. REDCap (www.project-redcap.org) is a secure, web-based application with controlled access designed to support data capture for research studies that provides audit trails for tracking data manipulation and export procedures.

All toxicities and adverse events will be recorded in the case report forms and graded for severity and cause according to the NCI Common Toxicity Criteria v4.0. Baseline signs and symptoms present at registration will be recorded as adverse events during the trial if they increase in NCI CTC v4.0 grade.

All events occurring during the conduct of the protocol and meeting the definition of a serious adverse event (SAE) under 21CFR 312.32 must be reported to the IRB. All SAEs, expected or unexpected, must be reported to the IND Office, regardless of attribution (within 5 working days of knowledge of the event). All life-threatening or fatal events, that are unexpected, and related to the study drug, must have a written report submitted within 24 h (next working day) of knowledge of the event to the Safety Project Manager in the IND Office.

SAEs will be captured from the time of the first protocol-specific intervention, until 30 days after the last study treatment/intervention, unless the participant withdraws consent, and must be followed until clinical recovery is complete, laboratory tests have returned to baseline, progression of the event has stabilized, or there has been acceptable resolution of the event. Any SAEs that occur after the 30 day time period that are related to the study treatment must be reported to the IND Office. This may include the development of a secondary malignancy.

Serious adverse events will be forwarded to FDA by the IND Sponsor (Safety Project Manager IND Office) according to 21CFR 312.32. The gene therapy reporting addendum (“Additional Reporting Form for Serious Adverse Events on Gene Therapy Trials”) will be included with each SAE submitted.

### Risk/benefit assessment

The primary objective of the study is to provide safety data on a personalized lymphoma DNA vaccine (scFv-CCL20 plasmid) administered by an intradermal needle-free injection device in small cohorts of asymptomatic LPL patients. Study risks include but are not limited to injection site reactions and transient flu-like symptoms. The primary endpoint of the study will be the determination of the MTD of scFv-CCL20. This information can be used to design future trials that seek to determine the efficacy of intradermal administration of this DNA vaccine in the treatment of asymptomatic LPL.

### Ethical approval

The study protocol was approved by the UTMDACC Institutional Review Board, and registered with ClinicalTrials.gov NCT01209871.

## Discussion

At the time of this writing, the first 6 patients have been enrolled and treated. Based on our IND-enabling animal toxicology studies with this DNA vaccine and past experience with other idiotype protein vaccines, we do not expect any Grade 3 or 4 toxicities. We anticipate that both doses will be well tolerated and the 2500 μg dose is likely to be chosen to evaluate the efficacy of the vaccine in a future phase 2 clinical trial.

The ideal patient population to benefit from this treatment strategy would be previously untreated LPL patients who are at high risk of early disease progression. While patient selection is outside the scope of this current phase 1 safety study, we fully anticipate incorporating eligibility criteria in a subsequent phase 2 clinical trial, which would select for such high-risk patients.

Finally, we expect that the absolute numbers of immunosuppressive cells in the pre-vaccine samples will likely correlate inversely with the quality and magnitude of the tumor/peptide-specific T cell responses and that such effector T-cell responses are likely to be higher in patients receiving the higher dose of the vaccine compared with the lower dose. We hypothesize that post-vaccination we will observe increased CD8^+^ (and CD4^+^) to FoxP3^+^ ratios and decreases in PD-1 and CTLA-4 expression by T cells. We expect to see an increased number and density of effector T cells interacting with LPL tumor cells, associated with vaccination. These results could inform the future development of new molecular biomarkers.

Further, the analysis of the secondary objectives will provide a preliminary readout of the biologic activity of the vaccine and will likely be hypothesis-generating. We expect to find imbalances in patients’ characteristics because this is an early phase, small size, and non-randomized trial. The data from these analyses will provide insight for, and assist in the design of, future studies even though the statistical power for the analysis will be limited, especially for smaller effects/differences. The immunogenicity data may also provide guidance on alternative vaccine formulations using other adjuvants or combinations with immune checkpoint inhibitors in future clinical trials, particularly if decreases in immune regulatory molecule expression are observed post-vaccination, or if robust effector immune responses are observed, but with only modest clinical responses.

## Abbreviations

ANA: antinuclear antibody
APCs: antigen-presenting cells
AST/ALT: aspartate transaminase alanine transaminase ratio
CCL20: CC chemokine ligand 20
CFR: Code of Federal Regulations
CTC: common toxicity criteria
CTLA-4: cytotoxic T-lymphocyte-associated protein 4
CTRC: Clinical Trials Research Center
DC: dendritic cells
DLT: dose-limiting toxicity
ECOG: Eastern Cooperative Oncology Group
ELISA: enzyme-linked immunosorbent assay
FITC: fluorescein isothiocyanate
FL: follicular lymphoma
GM-CSF: granulocyte macrophage-colony stimulating factor
HIPAA: Health Insurance Portability and Accountability Act
Id: idiotypic surface immunoglobulin
Ig: immunoglobulin
IND: investigational new drug
KLH: keyhole limpet hemocyanin
LPL: lymphoplasmacytic lymphoma
MCP3: monocyte chemotactic protein 3
MIP-3α: macrophage inflammatory protein-3α
MTD: maximum tolerated dose
NCI: National Cancer Institute
NSAIDs: non-steroidal anti-inflammatory drugs
PACE: platinum agent, doxorubicin, cyclophosphamide, etoposide
PBMC: peripheral blood mononuclear cells
REE: research eligibility evaluation
SAE: serious adverse event
scFv: single-chain variable fragment
UTMDACC: University of Texas M.D. Anderson Cancer Center
VH: heavy chain variable region
VL: light chain variable region
